# Antibacterial Activity of a Linolenic Acid Stigmasterol Ester Produced by Lipase-Mediated Transesterification

**DOI:** 10.4014/jmb.2410.10055

**Published:** 2025-02-14

**Authors:** Yeong A Kim, Hyung Kwoun Kim

**Affiliations:** Department of Biotechnology, The Catholic University of Korea, Bucheon 14662, Republic of Korea

**Keywords:** Linolenic acid stigmasterol ester, antibacterial activity, lipase, transesterification

## Abstract

Phytosterols are naturally found in lipid-rich plant foods and oils. These compounds exhibit various pharmacological effects, including anti-inflammatory, antimicrobial, antioxidant, and cholesterol-lowering properties. However, their industrial application has been limited due to their high melting points and poor solubility in both water and oil. Some unsaturated fatty acids can enhance phytosterols' oil solubility while exhibiting antimicrobial activities against various bacterial strains. In this study, we synthesized a linolenic acid stigmasterol ester (LASE) to improve oil and emulsion solubility of stigmasterol, a phytosterol known for its beneficial physiological effects in humans. LASE was synthesized with *Candida antarctica* lipase A through a transesterification reaction using stigmasterol and α-linolenic acid-rich linseed oil as substrates. The reaction was conducted at 50°C in isooctane with 20 mM of both stigmasterol and linseed oil. Following synthesis, LASE was purified using preparative liquid chromatography. The purified LASE demonstrated a 20-fold improvement in solubility in tricaprylin (TCN) compared to stigmasterol. Additionally, its antibacterial activities against specific food spoilage bacteria were confirmed using a TCN-based emulsion system.

## Introduction

Cholesterol is essential for various physiological functions. It is a key component of skin lipids, playing a crucial role in maintaining the skin barrier. It helps prevent moisture loss and blocks penetration of external substances [1, 2]. Additionally, cholesterol can be used as a valuable biomaterial in cosmetics and foods, serving as an emulsifier or oil additive [3, 4]. However, excessive accumulation of cholesterol in the body can lead to health issues such as hypercholesterolemia [5]. Phytosterol, a plant-derived sterol with a chemical structure similar to cholesterol, is a beneficial alternative as it does not pose the same clinical risks [6]. Found in plant oils and seeds, phytosterols are effective in lowering serum cholesterol levels, which is why they are added to various food products such as spreadable fats, yogurts, and milk [7, 8]. Moreover, phytosterols possess additional advantages including anticancer, anti-inflammatory, antifungal, and antibacterial properties [9, 10].

However, phytosterols have relatively high melting points and low solubility in oils, limiting their industrial applications [11]. For example, phytosterols have melting points of at least 135°C and a solubility of 1.24 g/100 ml in soybean oil [12]. To address this, some studies have focused on chemical modifications to enhance oil solubility of phytosterols for broader use [13]. Among these modifications, forming phytosterol fatty acid esters has been proven to be particularly effective in improving solubility in fats and oils [14]. Esterification with unsaturated fatty acids such as oleic acid, linoleic acid, and linolenic acid has significantly enhanced solubility of phytosterols [15].

Chemical synthesis of phytosterol esters requires complex separation and purification processes due to by-products and toxic chemical catalysts [16]. As a result, enzyme-catalyzed synthesis has gained increasing attention due to its advantages, including milder and more environmentally friendly reaction conditions, higher selectivity, and fewer by-products than chemical synthesis [17]. In enzyme-catalyzed reactions, esterification using free fatty acids produces water as a by-product, which can lead to hydrolysis and limit the equilibrium shift necessary for ester formation [18]. Meanwhile, when transesterification is performed, the reaction yield can be increased because water molecules are not generated [19]. That is, when plant oil is used as an acyl donor for a reaction, the target ester compound can be synthesized with a high efficiency through a transesterification reaction [20]. Recent efforts have used enzyme catalysts to synthesize phytosterol esters, addressing drawbacks of chemical methods [21]. However, studies on their detailed synthesis and characterization are limited, with studies on properties beyond cholesterol-lowering effects being scarce [12].

In this study, a linolenic acid stigmasterol ester (LASE) was synthesized using α-linolenic acid-rich linseed oil to improve both oil and emulsion solubility of stigmasterol, one of the most common plant sterols in human diet [22]. Furthermore, antibacterial activities of LASE against four food spoilage bacteria, *Bacillus coagulans*, *Bacillus subtilis*, *Alcaligenes faecalis*, and *Pseudomonas fluorescens* [23-25], were evaluated.

## Materials and Methods

### Materials

Acetonitrile, ethanol, cyclohexane, heptane, hexane, isooctane, toluene, and Tween 40 were purchased from Samchun Pure Chemical Co. (Republic of Korea). Tricaprylin (TCN), chloroform, LB broth media, and linseed oil were purchased from Sigma-Aldrich Co. (USA). Linolenic acid and stigmasterol were purchased from Tokyo Chemical Industry Co. (Japan). LB agar medium was purchased from Becton, Dickinson and Company (USA).

### Enzymes

*Candida antarctica* lipase B immobilized on acrylic resin (CalB, ≥ 5000 TBU/g) and *Candida rugosa* lipase immobilized on immobead 150 (CRL, ≥ 100 TBU/g) were purchased from Sigma-Aldrich Co. *Candida antarctica* lipase A immobilized on immobead 150 (CalA, ≥ 2000 TBU/g) was purchased from Apollo Scientific Co. (UK). *Proteus vulgaris* K80 lipase [26] was expressed in *Escherichia coli* BL21 (DE3). Recombinant K80 lipase in cell-free extract was immobilized onto methacrylate divinylbenzene bead (K80, 50 U/g) [27]. One TBU corresponds to the amount of enzyme that liberates one μmol butyric acid per min from tributyrin. One unit (U) of the enzyme corresponds to the amount that catalyzes the formation of one μmol of ethyl palmitate from p-nitrophenyl palmitate and ethanol through transesterification.

### Bacterial Strains

*Bacillus coagulans* (KCCM 11715) and *Pseudomonas fluorescens* (KCCM 41443) were purchased from Korea Culture Center of Microorganisms (KCCM, Republic of Korea). *Alcaligenes faecalis* (KCTC 2678) and *Bacillus subtilis* (KCTC 2189) were purchased from the Korea Collection for Type Cultures (KCTC, Republic of Korea).

### Optimization of Transesterification Reaction

To find an optimal reaction solvent, 20 mM stigmasterol, 40 mM linseed oil, and 20 mg of CalA were added to 1 ml of solvent (toluene, cyclohexane, hexane, heptane, and isooctane) to prepare a reaction mixture. The transesterification reaction was performed at 50°C in a shaking incubator at 210 rpm for 7 h and analyzed by HPLC after the reaction.

To find the optimal reaction temperature, 20 mM stigmasterol, 40 mM linseed oil, and 20 mg of CalA were added to 1 ml of isooctane to prepare a reaction mixture. The reaction was performed in a shaking incubator at 210 rpm for 7 h at various temperatures (30, 40, 50, and 60°C).

To find the optimal substrate molar ratio, the reaction mixture was prepared by adding 20 mM stigmasterol, different concentrations (10, 15, 20, and 25 mM) of linseed oil, and 20 mg CalA to 1 ml of isooctane. The reaction was performed at 50°C in a shaking incubator at 210 rpm for 7 h.

To find the most effective lipase, the reaction mixture was prepared by adding 20 mM stigmasterol, 20 mM linseed oil, and 20 mg lipase (CalA, CalB, CRL, and K80 lipase) to 1 ml of isooctane. The reaction was performed at 50°C in a shaking incubator at 210 rpm for 7 h.

To confirm the amount of LASE synthesized under the previously determined optimized conditions, the reaction was performed for 8 h and the concentration of LASE was measured by HPLC with a time course. The conversion yield was calculated using the following equation based on reduction of stigmasterol:

Conversion yield (%) = ((A_S0_-A_S_)/ A_S0_) × 100

Where A_S0_ and A_S_ were peak areas of stigmasterol before and after the reaction, respectively.

### HPLC Analysis

The transesterification reaction was analyzed using an HPLC (Agilent 1100 series, Agilent, USA). The reaction mixture was diluted 10-fold with ethanol and 10 μl was injected into an HPLC equipped with a reversed-phase C18 column (Cogent e-Column C18, 100 Å, 5 μm, 250 mm × 4.6 mm, MicroSolv Technology, USA). The mobile phase consisted of ethanol/acetonitrile (85:15, v/v). The flow rate was maintained at 1 ml/min. The temperature of the column oven was set to 45°C. The peak was detected at a wavelength of 210 nm. The external standard method was used for quantifying stigmasterol and LASE. A quantitative curve of stigmasterol was obtained using different concentrations (0.05–2.5 mM) of the standard solution. LASE was quantified by comparing peak areas with those of stigmasterol.

### Purification of LASE

The enzyme reaction was performed at 50°C for 12 h after adding 50 mM stigmasterol, 50 mM linseed oil, and 50 mg of CalA to 5 ml isooctane. Purification was performed using a preparative liquid chromatography (Prep-LC) (Waters, USA) equipped with a C18 column (XBridge BEH C18 OBD prep column, 130 Å, 5 μm, 50 mm × 250 mm, Waters). Then 4 ml of the sample was injected. The mobile phase consisted of ethanol/acetonitrile (75:25, v/v) at a flow rate of 70 ml/min. The sample was analyzed at 210 nm. LASE eluted in 20–25 min was collected. The solvent was completely evaporated using a rotary evaporator (N-1200A, EYELA, Japan). A concentrated sample was recovered using 5 ml of chloroform.

The purity of the purified LASE was tested with an analytical HPLC method. NMR spectra were obtained using an Avance III 300 MHz (Bruker BioSciences Corp., USA) in deuterochloroform (CDCl_3_).

### Preparation of LASE Emulsion

Stigmasterol, linseed oil, and LASE were each mixed with TCN to make a concentration of 400 mM, dissolved in a shaking incubator at 50°C for 1 h, and cooled to 25°C for 1 h. The undissolved portion was filtered through a filter (Nylon, 0.22 μm). The concentration of each sample was measured by HPLC. TCN containing the sample was mixed with sterile distilled water at a ratio of 1:1 (v/v). Tween 40 was added at a concentration of 8%. The mixture was sonicated on ice (5 min, 70% amplitude, pulse on for 2 sec, pulse off for 3 sec, VCX 130, Sonics & Materials Inc., USA) to make emulsions.

To determine the antibacterial activity of LASE according to its concentration, LASE was passed through a filter and diluted to various concentrations by adding TCN. An emulsion was prepared using the same method as described above.

### Antibacterial Activity of LASE

Antibacterial activities of the emulsion containing each sample against food spoilage bacteria were investigated. *B. coagulans*, *B. subtilis*, *A. faecalis*, and *P. fluorescens* were inoculated into 4 ml LB broth. *B. subtilis* and *P. fluorescens* were cultured at 30°C and *B. coagulans* and *A. faecalis* were cultured at 37°C for 16 h in a shaking incubator. The culture was diluted to 3 × 10^6^ colony forming units (CFU) per ml. Then 10 μl of the bacterial dilution was mixed with 50 μl of the emulsion and 40 μl of LB broth. The mixture was cultured at each incubation temperature for 24 h. The number of viable bacteria was measured by counting the CFU.

Antibacterial activities of emulsions containing various concentrations of LASE was investigated. *B. coagulans* and *A. faecalis* were cultured in a shaking incubator at 37°C for 16 h. Each culture was then diluted to 3 × 10^6^ CFU/ml. For *B. coagulans*, 10 μl of the bacterial dilution was mixed with 50 μl of LASE emulsion (0.23–2.3 mM) and 40 μl of LB broth. For *A. faecalis*, 10 μl of the bacterial dilution was mixed with 50 μl of LASE emulsion (0.023–0.23 mM) and 40 μl of LB broth. All mixtures were incubated at 37°C for 24 h and the number of viable bacteria was determined by counting CFU.

## Results and Discussion

### Synthesis of LASE

**Fig. 1:**
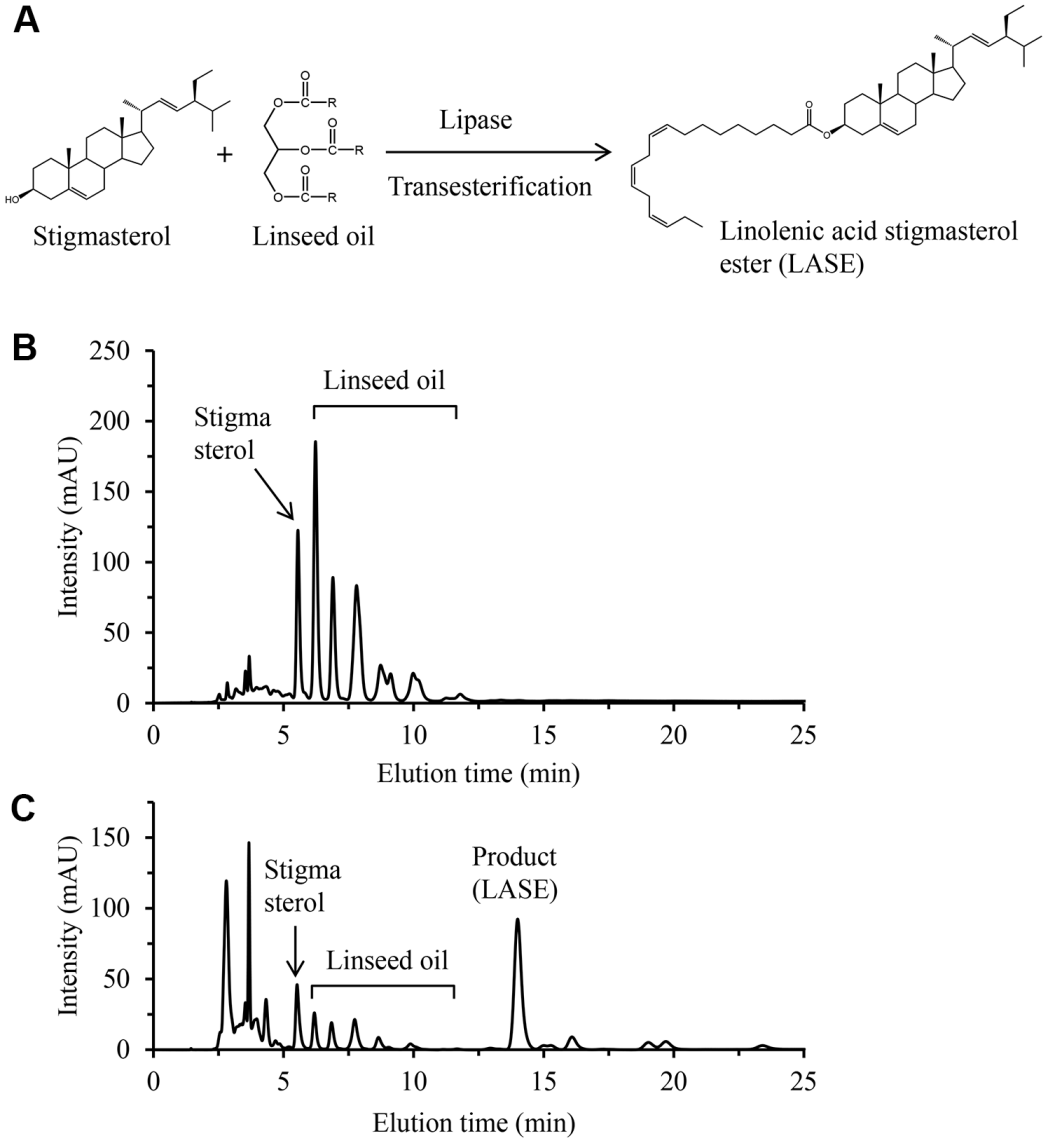
Synthesis of LASE. (**A**) Scheme of lipase-mediated transesterification of stigmasterol and linseed oil. (**B**) HPLC chromatogram of the reaction mixture at 0 h. (**C**) HPLC chromatogram of the reaction mixture at 7 h.

We synthesized LASE via lipase-mediated transesterification using linseed oil to enhance the solubility of stigmasterol in oils and emulsions (Fig. 1A). Linseed oil has the following fatty acid compositions: 4–5% palmitic acid, 2–3% stearic acid, 6–7% oleic acid, 12–18% linoleic acid, and 65–71% α-linolenic acid. Thus, this plant oil is a suitable source of α-linolenic acid [28]. To maximize LASE yield, four reaction parameters were optimized:

enzyme, solvent, substrate molar ratio, and temperature. Following an enzymatic reaction, the reaction mixture was analyzed by HPLC to confirm LASE production. Since a standard for LASE was not commercially available, the elution time was verified using LASE synthesized directly from esterification of stigmasterol and α-linolenic acid as a reference (Fig. S1). Stigmasterol and LASE were eluted at 5.5 and 14 min, respectively (Fig. 1B and 1C).

**Fig. 2:**
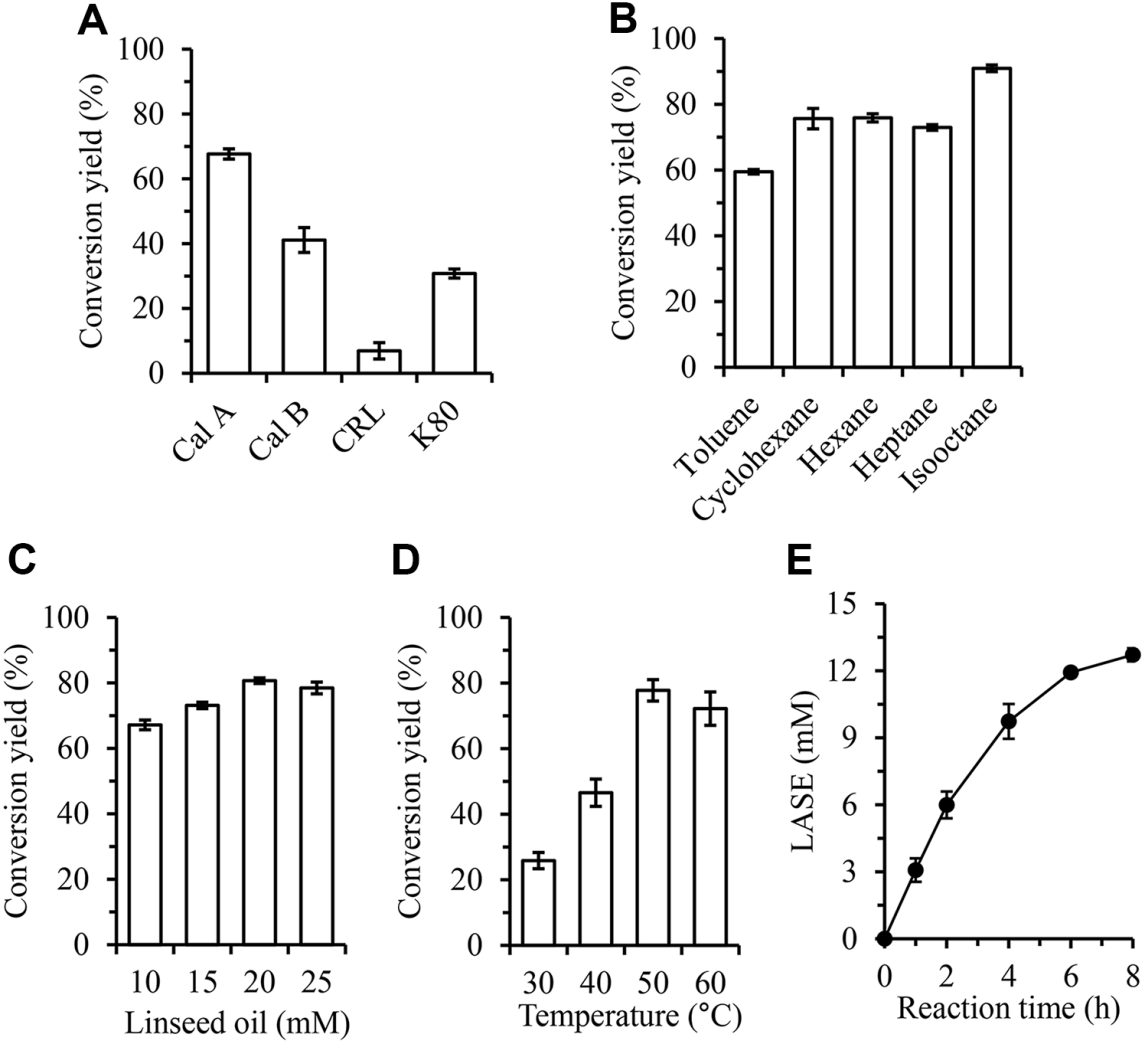
Optimization of lipase-mediated transesterification using stigmasterol and linseed oil. (**A**) Conversion yield using various lipases. Reactions were performed using 20 mM stigmasterol, 40 mM linseed oil, and 20 mg immobilized lipase in 1 ml isooctane for 7 h at 50°C. (**B**) Effects of organic solvents on transesterification. Reactions were performed using 20 mM stigmasterol, 40 mM linseed oil, and 20 mg of CalA in 1 ml organic solvent for 7 h at 50°C. (**C**) Effect of linseed oil amount on transesterification. Reactions were performed using 20 mM stigmasterol, 10–25 mM linseed oil, and 20 mg of CalA in 1 ml isooctane for 7 h at 50°C. (**D**) Effect of temperature on transesterification. Reactions were performed using 20 mM stigmasterol, 40 mM linseed oil, and 20 mg of CalA in 1 ml isooctane for 7 h. (**E**) Reactions were performed using 20 mM stigmasterol, 20 mM linseed oil, and 20 mg CalA in 1 ml isooctane.

We evaluated conversion yields with four lipases (CalA, CalB, CRL, and K80) to identify the most suitable enzyme for the transesterification reaction using stigmasterol and linseed oil. Using equal amounts of each enzyme (by weight), CalA achieved the highest conversion yield at 68%, followed by CalB at 41%, K80 at 31%, and CRL at 7% (Fig. 2A). CalA is a lipase with a narrow tunnel site that can accommodate acyl groups and a wide alcohol binding site [29]. It is suitable for accommodating complex alcohols such as phytosterols. It has shown high conversion yields in previous studies [30]. As CalA demonstrated a significantly higher yield under given conditions than the other three lipases tested, it was chosen for the synthesis of LASE.

The solvent choice is a critical factor in enzyme-mediated reactions for synthesizing plant sterol esters [31]. Solvents not only can facilitate mass transfer, but also can significantly influence lipase activity and stability by affecting the enzyme's structure and water activity [32]. To identify the optimal solvent for achieving the highest conversion yield, we tested five nonpolar solvents: toluene (log P 2.7), cyclohexane (log P 3.4), hexane (log P 3.9), heptane (log P 4.7), and isooctane (log P 4.8) [20]. Using CalA, isooctane provided the highest conversion yield of 91%, followed by cyclohexane and hexane with a yield of 76% for both, heptane with a yield of 73%, and toluene with the lowest yield of 59% (Fig. 2B). Previous studies have indicated that lipase activity diminishes as the log P of the solvent decreases [33]. Solvents with a high polarity (low log P values) can rapidly remove the necessary water from the lipase molecule, leading to enzyme inactivation [34]. Isooctane with its high hydrophobicity could allow for effective transesterification while maintaining enzyme activity.

To investigate the effect of stigmasterol-to-linseed oil molar ratio on conversion yield, we conducted the reaction with a fixed stigmasterol concentration of 20 mM and varied the linseed oil concentration from 10 mM to 25 mM. As the linseed oil concentration increased from 10 mM to 20 mM, the conversion yield increased to 81%. However, increasing the oil concentration to 25 mM did not further improve the yield (Fig. 2C). Given that each molecule of vegetable oil contains three fatty acids, a 20 mM concentration of stigmasterol and 20 mM linseed oil could provide three fatty acids per stigmasterol molecule. Considering both economic factors and conversion yield, subsequent reactions were carried out using 20 mM stigmasterol and 20 mM linseed oil.

Reaction temperature can influence enzyme activity, stability, and substrate solubility. Typically, as temperature increases, substrate solubility and lipase activity also increase whereas lipase stability decreases [12]. In this experiment, we examined CalA-mediated transesterification reaction across a temperature range of 30–60°C. We observed that the conversion yield peaked at approximately 78% for temperatures above 50°C (Fig. 2D). Conversely, at 30°C, the yield was the lowest at 26%, likely due to reduced substrate solubility and enzyme activity. Therefore, all subsequent experiments were conducted at 50°C.

Since the conversion yield was calculated based on substrate reduction, it was not possible to determine the exact amount of LASE synthesized. To quantify the LASE production under optimized conditions, we conducted the reaction and measured LASE concentration at various time points. The concentration of LASE increased rapidly during the first 4 h. However, extending the reaction time beyond 6 h did not result in a significant increase in its concentration, with the LASE concentration reaching 13 mM after 8 h (Fig. 2E). Thus, an optimal reaction time of 8 h was chosen for LASE production.

### Purification and Structural Analysis of LASE

**Fig. 3:**
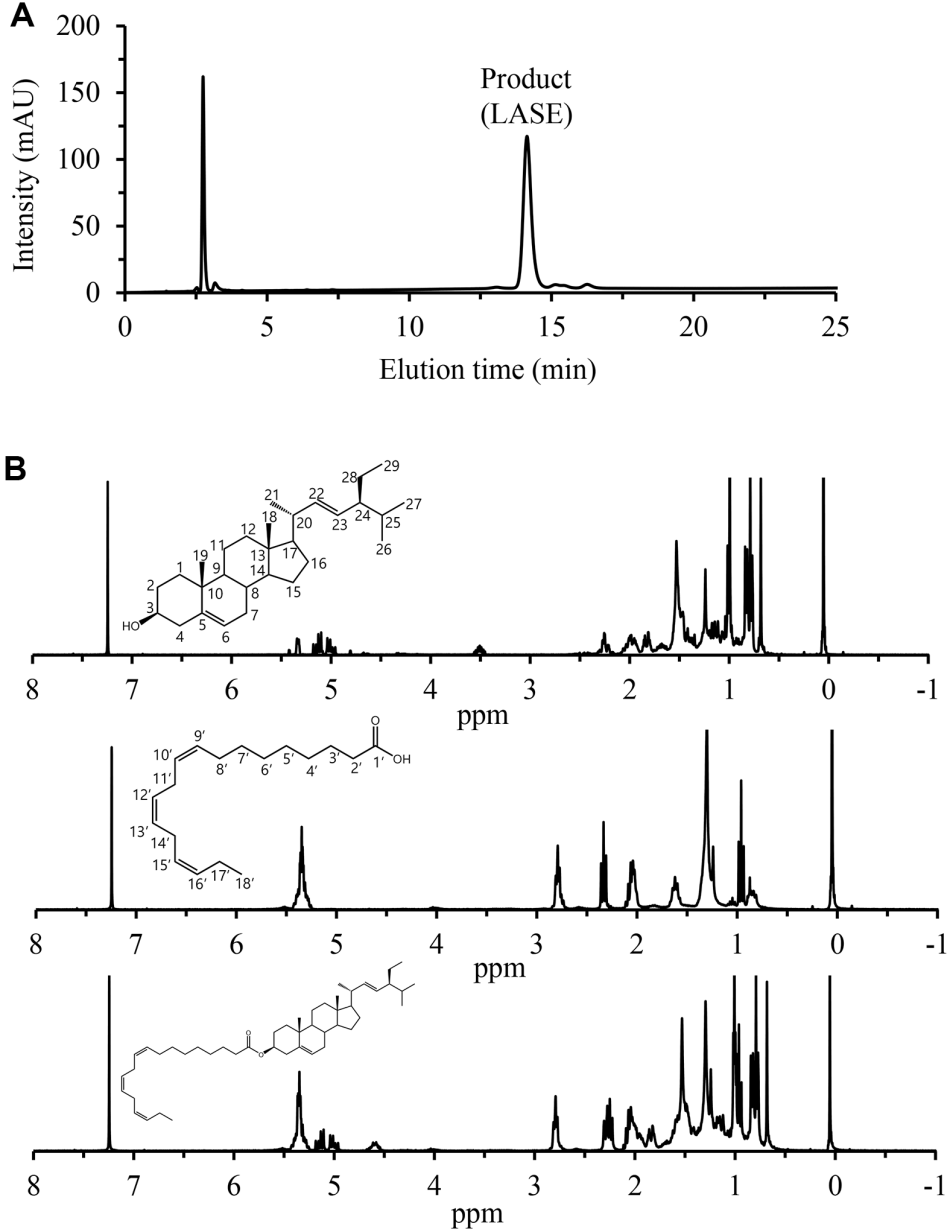
Purification and ^1^H-NMR analysis of LASE. (**A**) Purified LASE analyzed by HPLC. (**B**) ^1^H-NMR spectra of stigmasterol, linseed oil, and purified LASE.

After isolating LASE using Prep-LC, HPLC was performed to verify the purity of LASE. It was confirmed that all substrates and other impurity peaks observed in the reaction solution were removed (Fig. 3A).

Additionally, stigmasterol, linolenic acid, and purified LASE were analyzed by NMR, with its spectra shown in Fig. 3B. Chemical shifts observed in the ^1^H-NMR spectra confirmed the synthesis of LASE. Consistent with a previous report [35], multiple methylene hydrogen absorption signals appeared between 0.5 and 2.5 ppm, reflecting the complexity of the chemical environment. The 3-CH resonance of LASE at 4.6 ppm exhibited a 1.1 ppm downfield shift from stigmasterol’s resonance at 3.5 ppm, indicating the presence of an ester bond [36]. Additionally, the increase in hydrogen atoms at 5.3 ppm suggested the involvement of linolenic acid's 9'-CH, 10'-CH, 12'-CH, 13'-CH, 15'-CH, and 16'-CH, while the peak at 2.8 ppm corresponds to linolenic acid’s 11'-CH_2_and 14'-CH_2_ [37]. This analysis confirmed successful esterification of stigmasterol with linolenic acid and validated the structure of LASE.

### Antibacterial Activity of LASE

We prepared emulsions by adding stigmasterol, linseed oil, and LASE at a concentration of 400 mM each to TCN, followed by filtration to collect dissolved fractions. After adding Tween 40 and water, the mixture was then ultrasonicated to form emulsions. To evaluate the antibacterial activity of LASE, emulsions were prepared using Tween 40 and TCN, which had fatty acid compositions distinct from LASE. Specifically, Tween 40 contains palmitic acid and TCN contains caprylic acid, differing from linolenic acid in LASE.

Antibacterial activity was assessed by mixing the emulsion with bacterial culture medium at a 1:1 ratio (v/v) and incubating the mixture for 24 h. Due to differences in solubility, actual concentrations of stigmasterol, linseed oil, and LASE were 4.5 mM, 92 mM, and 92 mM, respectively (Table S1). As such, the solubility of LASE in TCN was 20 times greater than that of stigmasterol. Therefore, we expected that the LASE emulsion would have greater antibacterial activity than the stigmasterol emulsion. Given the turbidity of emulsions, absorbance-based cell growth measurements were not feasible. Instead, colony-forming units (CFUs) were counted to evaluate antibacterial effects.

**Fig. 4:**
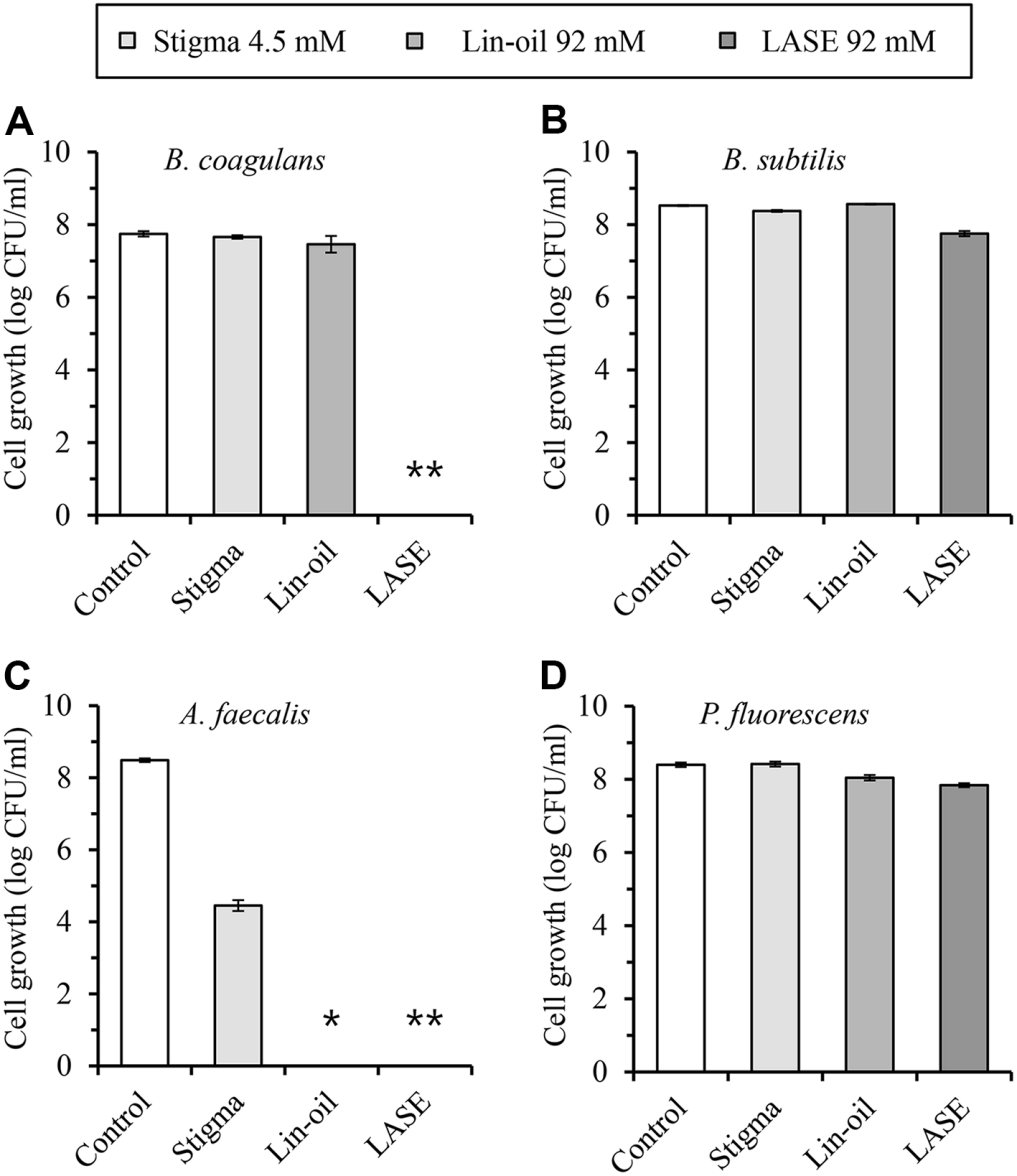
Antibacterial activities of compounds through an emulsion system. Antibacterial activities of control, stigmasterol, linseed oil, and LASE emulsions were measured by counting colony forming units (CFUs). The control consisted of an empty emulsion without adding any test compounds. (**A**) *B. coagulans*, (**B**) *B. subtilis*, (**C**) *A. faecalis*, (**D**) *P. fluorescens*. Symbols * and ** meant that no colony was formed.

When exposed to a control (empty) emulsion, four bacterial strains—*B. coagulans*, *B. subtilis*, *A. faecalis*, and *P. fluorescens*-exhibited similar CFU counts, confirming that Tween 40 and TCN did not affect bacterial growth. Stigmasterol (4.5 mM) emulsion partially reduced the CFU of *A. faecalis*, while linseed oil (92 mM) emulsion completely inhibited its growth. Notably, an LASE emulsion at 92 mM completely reduced CFUs of both *A. faecalis* and *B. coagulans* (Fig. 4). The antibacterial spectrum of LASE was broadened by combining sterol and unsaturated fatty acid. Observed activities of LASE against both Gram-positive (*B. coagulans*) and Gram-negative (*A. faecalis*) bacteria suggest its potential for applications as an antibacterial agent.

### Concentration-Dependent Antibacterial Activity of LASE

**Fig. 5:**
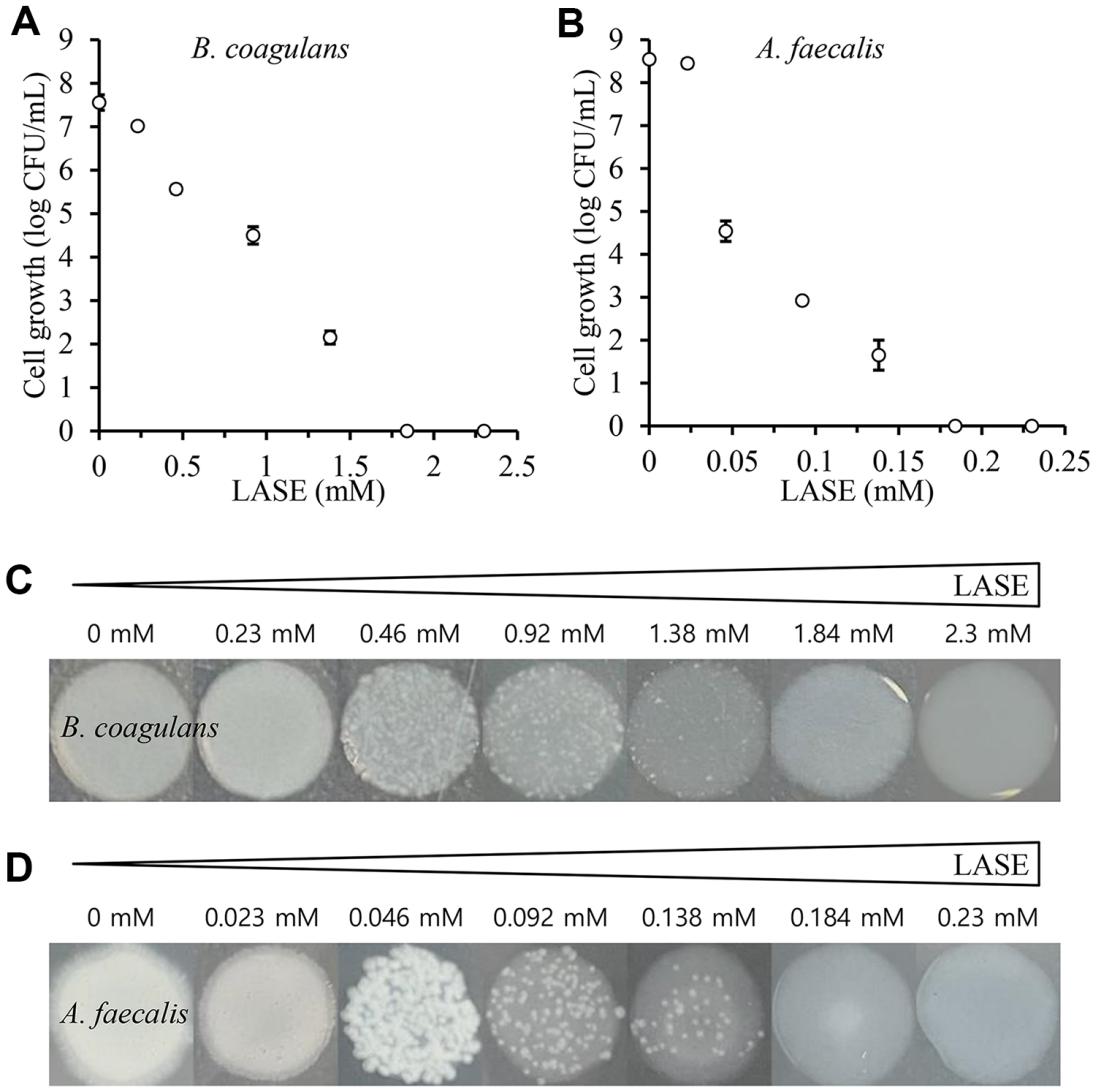
Concentration-dependent antibacterial activity of LASE emulsion. (**A**) CFUs of *B. coagulans*, (**B**) CFUs of *A. faecalis*, (**C**) Growth of *B. coagulans* after spot inoculation on agar plates, and (**D**) Growth of *A. faecalis* after spot inoculation on agar plates.

Since the LASE emulsion exhibited antibacterial activities against *B. coagulans* and *A. faecalis* at a relatively high concentration (92 mM), additional experiments were conducted to assess its effects at lower concentrations. Emulsions containing LASE at concentrations ranging from 0 to 2.3 mM for *B. coagulans* and 0 to 0.23 mM for *A. faecalis* were tested. For *B. coagulans*, approximately 70% CFU reduction was observed with 1.38 mM LASE emulsion and complete inhibition was observed with 1.84 mM LASE emulsion (Fig. 5A and 5C). In contrast, *A. faecalis* showed complete CFU inhibition in the presence of just 0.184 mM LASE emulsion (Fig. 5B and 5D). These findings demonstrate a concentration-dependent inhibitory effect of LASE on both bacterial strains and highlight a stronger antibacterial activity of LASE than stigmasterol.

This enhanced activity of LASE might result from a synergistic interaction between antibacterial properties of α-linolenic acid and stigmasterol. Previous studies have shown that stigmasterol can inhibit protein synthesis [38]. The antimicrobial potential of linseed oil has been attributed to polyunsaturated fatty acids, which can increase membrane fluidity and disrupt bacterial cell membranes [39]. The combination of these mechanisms may explain the observed synergistic effect of LASE. Further investigation is necessary to clarify more detailed mechanisms of its antimicrobial action.

This study confirmed that a novel stigmasterol fatty ester synthesized not only exhibited improved solubility in nonpolar environments, but also demonstrated an expanded antibacterial spectrum and increased efficacy. LASE is expected to serve as a valuable material for applications in cosmetics and food industries.

## Supplemental Materials

Supplementary data for this paper are available on-line only at http://jmb.or.kr.
