# Supplementary material for: Antibacterial Activity of a Linolenic Acid Stigmasterol Ester Produced by Lipase-Mediated Transesterification
Source: J Microbiol Biotechnol. 2025 Feb 14;35:e2410055. doi: 10.4014/jmb.2410.10055 (PMC11876018; doi:10.4014/jmb.2410.10055)
Supplement: Supplementary file 1 [file jmb-35-e2410055-supple.pdf]

J. Microbiol. Biotechnol. 2025. 35: e2410055
https://doi.org/10.4014/jmb.2410.10055

Supplementary Table S1. Concentration of substrates and LASE in emulsions

Substances	Concentration in TCN (mM)	Concentration after emulsion preparation (mM)	Final applied concentration (mM)
Stigmasterol	20	9	4.5
Linseed oil	400	184	92
LASE	400	184	92

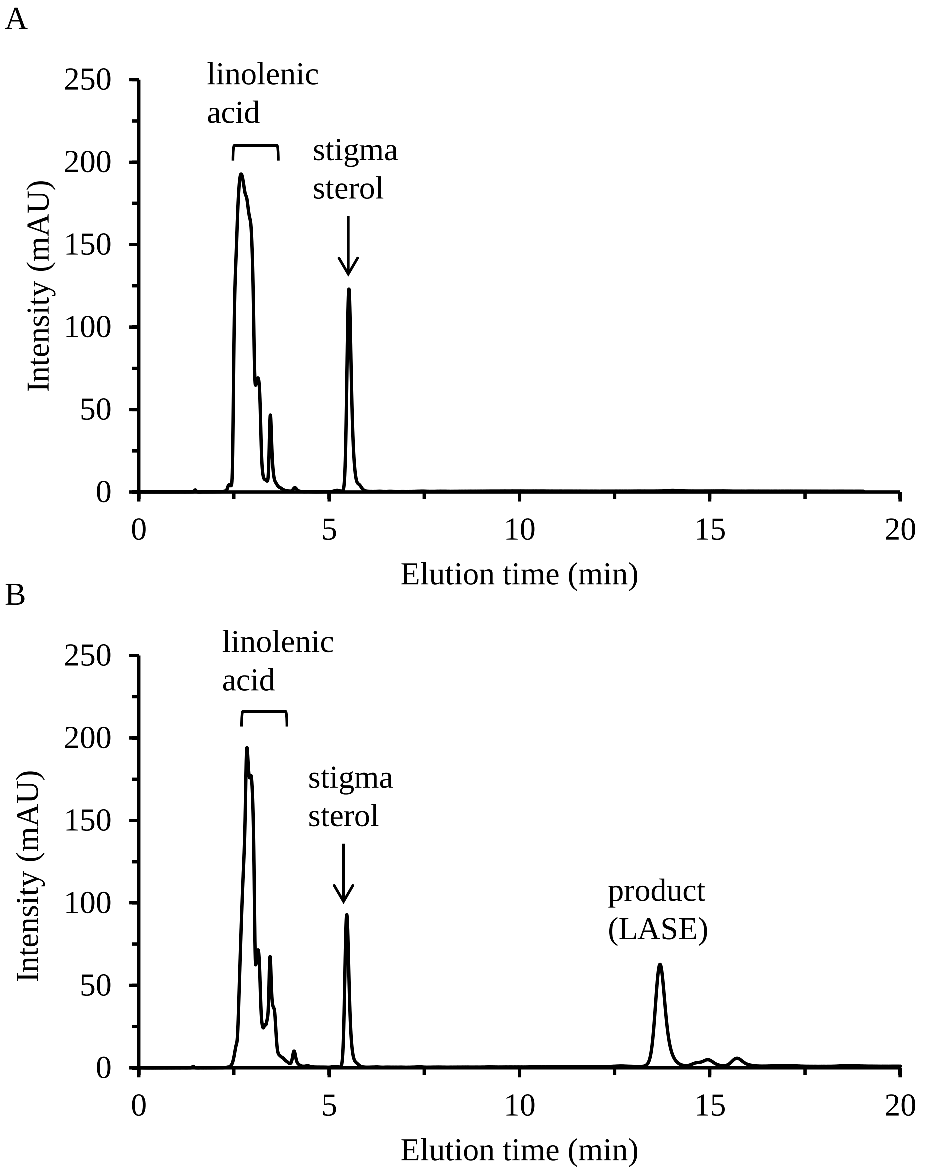

Supplementary Fig. S1. Synthesis of LASE by lipase-mediated esterification using stigmasterol and linolenic acid. Reactions were performed using 20 mM stigmasterol, 40 mM linolenic acid, and 20 mg CalA in 1 ml isooctane for 12 h at 40 °C. (A) HPLC chromatogram of the reaction mixture at 0 h. (B) HPLC chromatogram of the reaction mixture at 12 h.
